# Strategic planning for saving the lives of mothers, newborns and children and preventing stillbirths in KwaZulu-Natal province South Africa: modelling using the Lives Saved Tool (LiST)

**DOI:** 10.1186/s12889-015-2661-x

**Published:** 2016-01-19

**Authors:** Shelley-Ann McGee, Lumbwe Chola, Aviva Tugendhaft, Victoria Mubaiwa, Neil Moran, Neil McKerrow, Leonard Kamugisha, Karen Hofman

**Affiliations:** 1 Priority Cost-Effective Lessons for System Strengthening South Africa (PRICELESS SA), Medical Research Council/Wits Rural Public Health and Health Transitions Research Unit (Agincourt), Wits School of Public Health, Faculty of Health Sciences, University of the Witwatersrand, Wits Education Campus, 27 St Andrews Road, Parktown 2193, Johannesburg, South Africa; 2 Department of Health, Province of KwaZulu-Natal Pietermaritzburg, South Africa; 3 Department of Obstetrics and Gynaecology, Nelson R Mandela School of Medicine, University of KwaZulu-Natal, Durban, South Africa; 4 Department of Paediatrics and Child Health, Nelson R Mandela School of Medicine, University of KwaZulu-Natal, Durban, South Africa; 5 United Nations Population Fund (UNFPA), Pretoria, South Africa

**Keywords:** Mortality, Contraception, Maternal health, Stillbirths, Child health, Cost analysis, South Africa

## Abstract

**Background:**

KwaZulu-Natal province in South Africa has the largest population of children under the age of five and experiences the highest number of child births per annum in the country. Its population has also been ravaged by the dual epidemics of HIV and TB and it has struggled to meet targets for maternal and child mortality. In South Africa’s federal system, provinces have decision-making power on the prioritization and allocation of resources within their jurisdiction. As part of strategic planning for 2015–2019, KwaZulu-Natal provincial authorities requested an assessment of current mortality levels in the province and identification and costing of priority interventions for saving additional maternal, newborn and child lives, as well as preventing stillbirths in the province.

**Methods:**

The Lives Saved Tool (LiST) was used to determine the set of interventions, which could save the most additional maternal and child lives and prevent stillbirths from 2015–2019, and the costs of these. The impact of family planning was assessed using two scenarios by increasing baseline coverage of modern contraception by 0.5 percentage points or 1 percentage point per annum.

**Results:**

A total of 7,043 additional child and 297 additional maternal lives could be saved, and 2,000 stillbirths could be prevented over five years. Seventeen interventions account for 75 % of additional lives saved. Increasing family planning contributes to a further reduction of up to 137 maternal and 3,168 child deaths. The set of priority interventions scaled up to achievable levels, with no increase in contraception would require an additional US$91 million over five years or US$1.72 per capita population per year. By increasing contraceptive prevalence by one percentage point per year, overall costs to scale up to achievable coverage package, decrease by US$24 million over five years.

**Conclusion:**

Focused attention on a set of key interventions could have a significant impact on averting stillbirths and maternal and neonatal mortality in KwaZulu-Natal. Concerted effort to prioritize family planning will save more lives overall and has the potential to decrease costs in other areas of maternal and child care.

**Electronic supplementary material:**

The online version of this article (doi:10.1186/s12889-015-2661-x) contains supplementary material, which is available to authorized users.

## Background

South Africa has experienced an acceleration in the reduction of child mortality since 2006 [1] and a reversal of an increasing trend in maternal mortality ratios [2]. By 2013, national estimates put under-5 mortality rate at 41 per 1,000 live births, infant mortality rate at 29 per 1,000 live births and the maternal mortality ratio at 197 per 100,000 live births [2]. Despite this progress, South Africa has not reached the Millennium Development Goals (MDGs) for maternal and child mortality in 2015, which are 20 per 1,000 live births, 18 per 1,000 live births and 38 per 100,000 live births for under-5, infant and maternal mortality respectively [3]. In 2013, the country sought to identify key interventions that would be most effective in saving additional lives of mothers, newborns and children, in the 2 years leading up to the MDG deadline [3]. The aim was to contribute to a national strategy to reduce maternal and child mortality by 2015 and ensure progress towards the sustainable development goals. In line with this national agenda, the KwaZulu-Natal (KZN) provincial health department sought to localize the analysis, to identify context specific interventions that could save the lives of mothers and children in KZN.

The federal provincial structures in South Africa place provinces in control of resource allocations within their own jurisdictions. The National Health Act requires provincial Departments of Health to develop their own strategic plans, which must conform with national health policy [4]. This provides the provinces with the structure to implement their own priorities, responsive to the needs of their particular populations. KZN is the epicenter of the HIV/AIDS epidemic in the country, with an estimated HIV prevalence of 16.9 %, versus the national estimate of 12.2 % [5].

In 2014, the KwaZulu-Natal Department of Health, Maternal and Child and Women’s Health Unit began its strategic planning cycle for 2015–2019. The National Treasury requires that provinces and departments develop strategic plans and annual performance plans that are led by the South African Presidency [6]. The Treasury framework recommends that the costs of initiatives be linked with results to ensure value for money.

As part of the planning process, the KZN province sought to identify the cost and impact of scaling up essential interventions that could save additional lives of mothers, newborns and children, as well as prevent stillbirths in the province. With appreciation for the national set of priorities which had been determined in the national MDG analysis [3], the KZN provincial authorities wanted to identify a priority set of interventions specific to their own demographics and burden of disease for 2015–2019.

A process was initiated to evaluate the current situation in the province, better understand the challenges faced in terms of maternal and child mortality and use an available policy development software tool to model the potential impact on maternal and child mortality that could be achieved over the strategic five year period.

This analysis aimed to identify and cost a set of interventions to be prioritized and scaled up by 2019 to reduce maternal, newborn and child deaths and prevent stillbirths in the province.

## Methods

### Study area

KwaZulu-Natal (KZN) province is the second most populous province in South Africa, and has the largest population of children under five years old with 1.3 million in 2014 [7] or 22 % of the children in the country. The province also has the largest population dependent on the public health sector for services [8]. KZN has been the most severely impacted by the country’s HIV/AIDS and tuberculosis epidemics. It is the province with the highest HIV prevalence in antenatal clients, at 37.4 % [9] and the highest incidence of tuberculosis in the general population, at 971 per 100,000 population in 2012 [10].

Since 2010, however, maternal and child health indicators have been improving. Maternal mortality in healthcare facilities in the province has decreased from a peak of 209 per 100,000 live births in 2010, down to 160 per 100,000 live births in 2012 [11, 12]. The figures are likely an underestimate, however, as recording and reporting on maternal deaths outside of healthcare facilities remains a challenge in the country [13]. Under five deaths in the province have decreased by about 46 % from 13,000 in 2007 to 7,000 in 2011 [14]. Having achieved considerable success in coverage of prevention of mother to child transmission of HIV (PMTCT) as well as improvement in access to antiretroviral treatment, the province is in a position to consider the potential impacts of other key interventions for saving the lives of mothers and children.

### Modelling software

The analysis was undertaken using the Lives Saved Tool (LiST) within the Spectrum software (Futures Institute, now Avenir Health, United States of America), which was developed to support decision making in the health sector [15, 16]. The Spectrum program consists of several modules which interact with one another to address a variety of issues in demography and population health. The demographic projection model (DemProj) forms the basis for any Spectrum projection and requires inputs on various determinants of population dynamics, including fertility rates, mortality rates and population age distribution. The modelling for maternal and child health impacts is done in the LiST, while the Family Planning (FamPlan) Module examines the impacts of changing contraceptive use prevalence and methods mix. FamPlan requires inputs on total fertility, and contraceptive prevalence, as well as the proximate determinants of fertility including the proportion of women of reproductive age married or in a sexual union, duration of post-partum insusceptibility, abortion rates and degree of pathological sterility in the population [16]. The analysis was conducted using Spectrum version 5.06.

The functionality of LiST has been described in detail in previous publications [17–19]. LiST is a linear mathematical model which describes fixed relationships between inputs, which are primarily levels of coverage for a set of interventions, and outputs in terms of changes in population level risk factors and causes of mortality in children, neonates, pregnant women and stillbirths [18]. LiST is capable of analyzing the impact of the scale up of multiple interventions, which may be effective in reducing risk and mortality from several causes. For example, one particular scenario modelled can calculate the impact of a package of interventions as they are scaled up from a baseline coverage level to forecast the impact of various projected levels of coverage over time.

LiST preloads national-level data for health status, mortality rates, and coverage of approximately 70 interventions, and their effectiveness in relation to specific causes of death in mothers, neonates and children under 5 years of age for a national level analysis [17, 20]. The Spectrum software allows for sub-national analyses to be constructed using a country-based model. In order to complete the province-specific analysis, a subnational population scenario was developed within a national level baseline model [3]. This involved collection of province-specific information on population numbers and growth rate, total fertility rates, contraceptive prevalence rate and HIV prevalence information for KZN province [5, 7, 9].

The LiST default settings do not carry all possible interventions, thus several additional interventions were included in the modelling, in response to existing policies and burden of disease. Given the significant burden of HIV and TB in the province, treatment of childhood tuberculosis [21], treatment of tuberculosis in pregnant women [22], as well as early detection and treatment of HIV in pregnant women were included. In response to the Free State province’s success in reducing maternal mortality with improved interfacility transport for obstetric emergencies, this was also included as a potentially impactful intervention [23]. In children between 1 and 4 years old, non-natural causes are responsible for 17.4 % of total deaths, with external sources of accidental injury as the source of 70 % of these non-natural deaths [24]. Thus treatment of childhood injuries was also included as an intervention to save child lives between the ages of 1 and 59 months [25].

These interventions were assigned to the causes of death which they could potentially affect. Intervention effectiveness was drawn from the literature and expert input from a national Millennium Development Goal Countdown analysis in LiST which was undertaken for South Africa in 2013 [3, 21, 23, 26]. Details of the effect sizes and affected fractions which were applied to the model are given in Additional file 1: Sheet 1.

Adequately modelling the provincial-level impact of different interventions required the following:

- A baseline assessment of demographic information, epidemiological detail (particularly on HIV and tuberculosis), rates and causes of maternal and child mortality and stillbirths and several other indicators contributing to the LiST Tool.
- Estimation of current (baseline) coverage of the LiST set of interventions.
- Extrapolating the impacts of different scenarios of coverage from baseline in 2014 to the end of the strategic period in 2019.
- Estimating the costs of the scenario to assist in budgeting and planning.

### Baseline analysis

Data to populate the Spectrum Suite were drawn from a number of sources available on HIV, maternal and child health indicators, under-5 and maternal deaths and stillbirths in the province, as well as estimates of the coverage levels of interventions in LiST but not routinely monitored or reported in South Africa.

Burden of disease estimates for 2011 for maternal, neonatal and child deaths were derived from the National Committee on Confidential Enquiries into Maternal Deaths (NCCEMD) data, and vital registration data [14, 27]. The causes of maternal, neonatal and under-5 deaths were adapted in consultation with the provincial clinical team to fit the causal categories provided by LiST. Mortality rates were determined for 2011 or the closest year.

Many of the interventions in LiST are not tracked on a consistent basis within the health monitoring systems. The baseline coverage for LiST indicators was determined using national data from the District Health Information System [28] where possible. Where estimates were not available from standard database sets, the provincial clinical and management team was asked to estimate the coverage for particular interventions, based on their experience and knowledge of the health services in the province.

This team consisted of the head of the maternal and child health programme in the province as well as two clinical specialists, one in obstetrics and gynaecology and the other in paediatrics. Consultation with the team was a deliberative process during which baseline coverage levels (Table 1) as well as the interventions, their meaning and application in the provincial context was discussed and explored.

**Table 1:** Baseline intervention coverage based and achievable levels for LiST interventions as adapted and modified by provincial experts (July 2014)

Intervention	Baseline reviewed by experts (KZN)	Achievable scale up by 2019 estimate
	2014 (%)	
Preconceptual options		
Folic acid supplementation/fortification	90	93
Safe abortion services	20	60
Post abortion case management	0	20
Ectopic pregnancy case management	0	0
Pregnancy interventions		
Antenatal care	53.9	65
TT - Tetanus toxoid vaccination	60	90
Syphilis detection and treatment	50	85
Nutritional		
Calcium supplementation	50	80
Micronutrient supplementation (multiple micronutrients + iron folate)		
Iron folate supplementation	80	90
Multiple micronutrient supplementation	60	75
Balanced energy supplementation	2	20
Case management		
Hypertensive disease case management	60	80
Diabetes case management	10	20
MgSO4 - Management of pre-eclampsia	70	90
FGR - Fetal growth restriction detection and management	10	25
Child birth		
Skilled birth attendance (SBA)	84.5	90
Facility delivery (clinic and hospital)	84.5	90
Level of delivery		
Home deliveries	7.2	5
Unassisted deliveries	7.2	5
Assisted deliveries at home (SBA)	0	0
Facility deliveries		
Essential care	55	33
BEmOC (Basic Emergency Obstetric Care)	2	10
CEmOC (Comprehensive Emergency Obstetric Care)	28	45
All deliveries		
Clean birth practices	80	90
Immediate assessment and stimulation	90	98
Labor and delivery management	93	98
Neonatal resuscitation	40	95
Antenatal corticosteroids for preterm labor	40	60
Antibiotics for pPRoM	75	90
MgSO4 management of eclampsia	90	95
AMTSL--active management of the third stage of labor	90	95
Induction of labor for pregnancies lasting 41+ weeks	20	45
Promotion of breastfeeding	80	90
Preventive options		
Postnatal care		
Preventive postnatal care	72	95
Thermal care	72	95
Clean postnatal practices	72	95
Chlorhexidine	0	0
Feeding and supplements		
Complementary feeding--education only	30	50
Complementary feeding--supplementation and education	30	50
Vitamin A supplementation	39	51
Zinc supplementation	0	0
WASH		
Improved water source	86	93.8
Water connection in the home	63.6	72.6
Improved sanitation - Utilization of latrines or toilets	53.2	57.6
Hand washing with soap	17	40
Hygienic disposal of children’s stools	40.5	50
Vaccines		
BCG	78	90
Polio	73	90
DPT	72	90
Hib	72	90
HepB	76	90
Pneumococcal	72	90
Rotavirus	72	90
Measles	78	90
Curative options		
Neonatal		
Maternal Sepsis case management	15	60
KMC - Kangaroo mother care	10	66
Case management of severe neonatal infection	50	90
Oral antibiotics	0	0
Injectable antibiotics	50	20
Full supportive care	50	80
Diarrhoea		
ORS - oral rehydration solution	40	70
Antibiotics - for treatment of dysentery	58	80
Zinc - for treatment of diarrhoea	0	0
Other infectious diseases		
Oral antibiotics: case management of pneumonia in children	73	90
Vitamin A - for treatment of measles	75	90
Antimalarials - Artemesin compounds for malaria	0	90
Therapeutic feeding - for severe wasting	0	50
Treatment for moderate acute malnutrition	0	10

**Fig. 1:**
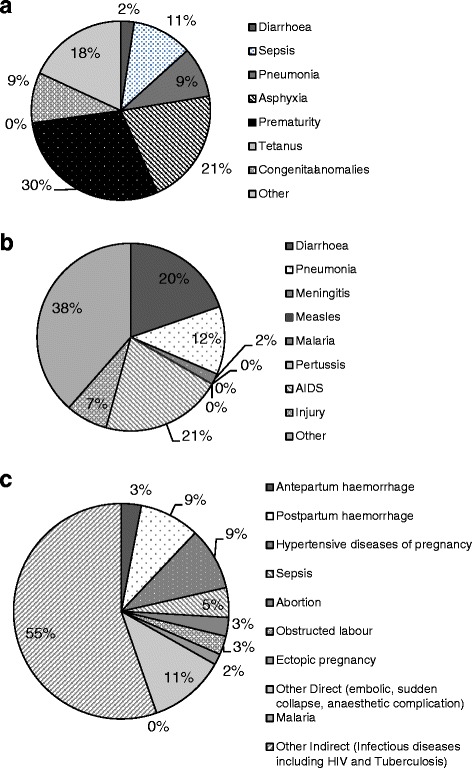
Proximate Causes of **a** newborn deaths, **b** deaths in children 1–59 months of age and **c** maternal deaths in KwaZulu-Natal in 2011. Source: Adapted from Stats SA and Vital Registration details of newborn deaths in 2010 and 2011 and the National Confidential Enquiries on Maternal Deaths Interim Report 2011 and 2012

Using 2011 as baseline, we used a provincial maternal mortality rate of 187 deaths per 100,000 live births [27], a neonatal mortality rate of 13.6/1,000 [29] and an under-5 mortality rate of 37.08/1,000 (estimated from vital statistics data, adjusted for underreporting using national level methods) (Additional file 1: Worksheet 2) [14]. Baseline stillbirth rate was 23/1 000 live births [28]. The causes of maternal mortality [27] and neonatal and child mortality [14] were adapted from their source estimates to fit the causal categories in LiST, and adjustments were validated by the clinical provincial team by comparison to nationally reported estimates [3] and provincial estimates where these were available [12, 14] (Fig. 1).

### Projection and analysis

The provincial team was also asked to project the level of coverage for each intervention that they estimated KZN would reach by 2019, if concerted effort were realistically applied and considering existing policy changes, resource inputs and observed local impacts of existing services (Table 1), i.e., the level of coverage attained by realistically scaling up essential interventions. In this paper, this level of coverage is referred to as ‘achievable coverage’.

The LiST was used to analyze the number of deaths of mothers, newborns and children and stillbirths that could be averted by scaling up all interventions in the model from their baseline coverage estimates to:

- A full coverage scenario (defined as all interventions increased to 95 % coverage) and
- An achievable coverage scenario (estimated achievable coverage varies per intervention)

The “full coverage” scenario aimed to show what would be possible in terms of reducing the numbers of deaths, if a universal coverage was achieved for all interventions, whereas the “achievable scenario” was considered to be what could be realistically achieved with the prevailing conditions in the province. The achievable scenario was also used for the subsequent costing exercise to inform provincial planning.

Family planning also contributes significantly in reducing the numbers of overall deaths of mothers, infants and children, and reducing the number of stillbirths. Averting unwanted pregnancies through improved family planning and birth spacing can potentially have a substantial impact on reductions in maternal and perinatal deaths [30]. Thus, in addition to the two base scenarios, two analyses were completed for contraceptive use prevalence (using the FamPlan Module of Spectrum):

- Contraceptive use increases by 0.5 percentage points per annum from baseline
- Contraceptive use increases by one percentage point per annum from baseline

The FamPlan method mix was determined at baseline for 2014 using information from the provincial DHIS, and main changes to the mix were an increase in the uptake of the newly introduced etonorgestrel implant, with ongoing decrease of the percentage use of intrauterine devices and injectable options – a trend which was already evident from the contraceptive utilization data (Additional file 1: Worksheet 3).

The effect of scaling up the maternal and child intervention coverage was modelled to estimate the deaths averted, overall and by each intervention.

### Costing methodology

Intervention costs were calculated from a provider perspective, using the costing module within the LiST. The module uses an ingredients approach to costing, based on four main components: personnel and labour; drugs and supplies; other recurrent costs and capital costs. The first year of costing was 2014.

Staff remuneration was based on current salary structures for healthcare workers in South Africa, as published, and adjusted for annual increases, at 5.6 % per annum [31]. For unit costs of medicines and supplies, the default values in LiST were used. These unit costs are based on international prices from UNICEF and the Management Sciences for Health International Drug Price Indicator Guide 2011 [32]. Unit costs in Spectrum for medicines and supplies were generally comparable to the South African prices of drugs and supplies on various national tenders in most instances, although changes were made specifically to the costs for family planning commodities, since the costs of the sub-dermal implant had decreased substantially [33].

Recurrent costs related to hospitalization and outpatient visits were not included. Recurrent costs include personnel training, gasoline, building rent, office supplies and promotional activities. These were outside of the scope of the analysis. Capital costs such as infrastructure expenditure were also excluded from the analysis. Thus the costing reflects purely the scaling up of the delivery of each intervention in terms of costs of staff time and costs of medicines and supplies.

To judge the relative additional number of lives saved against the relative additional costs incurred to scale up interventions, a ratio cost per life year saved was determined. This was done by multiplying the lives saved by the expected life expectancy for each life saved. The average provincial life expectancy in 2014 of 54.4 years [7] was used for child and neonatal deaths and stillbirths prevented. For maternal lives saved, we used the Reproductive-Aged Life Expectancy – RALE [34], estimated to be 27 years, or half of the average life expectancy at birth.

## Results

### Reduction in child newborn and maternal mortality and stillbirths by 2019

#### Full coverage (95 %)

The model estimated that if all interventions were to be scaled up to full coverage (95 %) from baseline by 2019, maternal mortality could be reduced by 50 % to 74/100,000. Neonatal and under-5 mortality both reduce by 46 % to 5.6/1,000 and 17.8/1,000 respectively.

At full coverage by 2019, 5 interventions would account for 81 % of additional maternal lives saved, 12 interventions would account for 70 % of additional child lives saved, and 3 interventions for 83 % of stillbirths prevented (Table 2). Labour and delivery management is an intervention highly ranked in saving both maternal and child lives, and preventing stillbirths. Promotion of breastfeeding and handwashing with soap, two community based interventions, could have a significant impact on saving child lives, accounting for nearly 20 % of child lives saved over 5 years.

**Table 2:** Ranked additional lives saved per LiST intervention by maternal and child lives saved and stillbirths prevented 2019 if fully scaled up (95 %) from baseline

Rank	Interventions saving maternal lives	Total lives saved	% of lives saved	Interventions saving child lives (0–60 months)	Total lives saved	% of lives saved	Interventions preventing stillbirths	Total lives saved	% of lives saved
1	Labour and delivery management^a^	146	25.4	Promotion of breastfeeding	1332	10.9	Labour and delivery management^a^	2132	47.1
2	Early detection and treatment of HIV (pregnant women)	137	23.8	Hand washing with soap	909	7.4	Syphilis detection and treatment	962	21.3
3	TB treatment in pregnant women	83	14.4	Labour and delivery management^a^	895	7.3	Foetal growth restriction detection and management	577	12.8
4	Safe abortion services	58	10.1	Water connection in the home	867	7.1	All other interventions (5)	785	17
5	Inter-facility transport	26	4.5	Antiretroviral Therapy (ART)	721	5.9			
6	All other interventions (9)	110	19	Antenatal corticosteroids for preterm labour	636	5.2			
7				Full supportive care for sepsis/pneumonia	609	5.0			
8				Pneumococcal Vaccine	598	4.9			
9				Therapeutic feeding - for severe wasting	552	4.5			
10				ORS - oral rehydration solution	540	4.4			
11				PMTCT - Prevention of mother to child transmission of HIV	461	3.8			
12				Improved sanitation - Utilization of latrines or toilets	458	3.7			
13				All other interventions (23)	3662	30			
	Maternal lives saved by top five interventions 2015-2019	450	81	Child lives saved by top 12 interventions 2015-2019	8578	70	Stillbirths prevented by top 3 interventions 2015-2019	3671	83
	Total maternal lives saved 2015-2019	560	100	Total child Lives saved 2015-2019	12240	100	Total stillbirths prevented 2015-2019	4456	100

^a^Common intervention

### Achievable coverage

The achievable coverage scenario demonstrated a reduction in maternal mortality of 26 % to 110/100,000 and 28 % for neonatal and under-5 mortality to 7.5/1,000 and 23.5/1,000.

This mortality rate decrease translates into the potential to save an additional 297 maternal, 2,300 neonatal and 4,800 one-month to five-year old lives cumulatively by 2019 as coverage levels are improved (Table 3).

**Table 3:** Ranked additional lives saved per LiST intervention by maternal and child lives saved and stillbirths prevented 2019 if fully scaled up to achievable levels from baseline

Rank	Interventions saving maternal lives	Total lives saved	% of lives saved	Interventions saving child lives (0–60 months)	Total lives saved	% of lives saved	Interventions preventing stillbirths	Total lives saved	% of lives saved
1	Early detection and treatment of HIV (pregnant women)	69	23.2	ORS - oral rehydration solution	877	12.5	Labour and delivery management^a^	779	37.9
2	Labour and Delivery Management^a^	53	17.8	Promotion of breastfeeding	655	9.3	Syphilis detection and treatment	764	37.2
3	TB treatment in pregnant women	43	14.4	Full supportive care for sepsis and pneumonia	559	7.9	All other interventions (5)	511	24.9
4	Safe abortion services	30	10	Handwashing with soap	434	6.1			
5	Maternal sepsis Case management	23	7.7	Oral Antibiotics: Case management of pneumonia in children	421	6.0			
6	All other interventions (9)	79	27	Therapeutic feeding for severe wasting	416	5.9			
7				Pneumococcal Vaccine	403	5.7			
8				Antiretroviral Therapy (ART)	362	5.1			
9				Neonatal resuscitation	350	5.0			
10				Labour and Delivery Management^a^	317	4.5			
11				Kangaroo Mother Care (KMC)	306	4.3			
12				PMTCT	288	4.1			
13				All other interventions (23)	1655	23.5			
	Maternal lives saved by top 5 interventions 2015-2019	218	74	Child lives saved by top 12 interventions 2015-2019	5388	77	Stillbirths prevented by top 3 interventions 2015-2019	1543	75
	Total maternal lives saved 2015-2019	297	100	Total child Lives saved 2015-2019	7 043	100	Total stillbirths prevented 2015-2019	2 054	100

^a^Common intervention

In the achievable coverage scenario, 5 interventions account for 74 % of additional maternal lives saved, 12 interventions account for 77 % of child lives saved and 2 interventions account for 75 % of stillbirths prevented. Labour and delivery management again features as an intervention common to saving lives in all three areas. In total, these 17 interventions could potentially save 5,388 additional child lives, 218 maternal lives and prevent an additional 1,543 stillbirths from 2015 to 2019 (Table 3). For saving maternal lives, early detection and treatment of HIV, labour and delivery management and TB treatment in pregnant women are the most important, given the high burden of maternal deaths from HIV and TB in the province and the relatively high mortality from hemorrhage. For children, coverage of several preventative measures was quite high at baseline, with the result that curative measures such as provision of oral rehydration solution and full supportive care for sepsis and pneumonia in neonates save a large number of additional lives. Promotion of breastfeeding can also have a significant impact on saving lives, if increased promotion is associated with increased exclusive breastfeeding behavior. Labour and delivery management remains the most important intervention in the prevention of stillbirths.

### Addition of increasing contraceptive prevalence

With the addition of improving contraceptive prevalence in parallel with achievable scale up of maternal and child interventions, the potential number of lives saved increases even further. When contraceptive prevalence increases by 0.5 percentage points per annum from 2015 to 2019, a further 69 maternal deaths, 1,452 child deaths and 1,135 stillbirths could be prevented (Additional file 2: Worksheet 4). If contraceptive prevalence increases by 1 percentage points per annum from 2015 to 2019, a further 137 maternal deaths, 3,168 child deaths and 2014 stillbirths could be prevented (Additional file 2: Worksheet 5).

### Costs for achievable coverage scenario

The baseline cost in 2014 for the total suite of LiST interventions for which costs were estimated was US$123 million (about US$11.62 per capita and the 2014 provincial population estimate of 10.69 million [7]).

Table 4 summarizes the costs expected over 5 years if the full list of interventions is scaled to achievable coverage. With no increases in contraceptive use, the total cost of these interventions would be an additional US$141 million over the five-year period, or approximately US$2.65 per capita per annum. Where contraceptive use is assumed to increase, the additional costs of contraceptive services increase from US$15.3 million with no contraceptive scale up, to US$20.1 million where contraceptive use prevalence increases by one percentage point per year. Despite this increase, overall costs for the scale up of services decreases from US$140.6 million to US$102.8 million. Thus improved contraceptive use is cost saving, by US$37.8 million, where all else remains equal.

**Table 4:** Projected incremental costs from 2015 to 2019 for all interventions scaled to achievable levels within three family planning scenarios

		Family planning does not increase	Family planning increases by 0.5 percentage points per annum	Family planning increases by 1 percentage points per annum
		Incremental costs over five years (US$)	Incremental costs over five years (US$)	Incremental costs over five years (US$)
	Intervention costs	125 272 810	102 437 129	82 645 306
	Family planning costs	15 290 627	17 694 164	20 113 685
Salary increases included at 5.6 % per annum	Overall cost	140 563 438	120 131 293	102 758 991
	Average Cost per annum	28 112 687	24 026 258	20 551 798
	Overall cost per capita per annum^a^	2.65	2.27	1.94

^a^Assuming population of 10.6 million people

Scaling up only the priority list of seventeen interventions would cost US$91 million over five years, or US$1.72 per capita per year (Table 5). Thus 64 % of potential expenditure can achieve about 75 % of the additional lives saved.

**Table 5:** Projected incremental costs from 2015 to 2019 for top 17 interventions scaled to achievable levels within three family planning scenarios

		Family planning does not increase	Family planning increases by 0.5 percentage points per annum	Family planning increases by 1 percentage points per annum
		Incremental costs over five years (US$)	Incremental costs over five years (US$)	Incremental costs over five years (US$)
	Intervention costs	75 573 421	60 992 608	46 468 465
	Family planning costs	15 410 296	17 694 164	20 113 685
Salary increases included at 5.6 % per annum	Overall cost	90 983 717	78 686 772	66 582 150
	Average Cost per annum	18 196 743	15 737 354	13 316 430
	Overall cost per capita per annum^a^	1.72	1.49	1.26

^a^Assuming population of 10.6 million people

When the contraceptive prevalence rate increases by 0.5 percentage points per annum, the overall cost increase to scale up the 17 priority interventions reduced, and an additional US$79 million would be required over the five years for these priority interventions (US$1.49 per capita per annum) (Table 5). Where contraceptive prevalence rate increased by 1 percentage point per annum, this cost saving effect was more pronounced, resulting in total incremental costs of US$67 million (US$1.26 per capita per annum). Thus in the prioritized interventions, improving contraceptive prevalence could save US$24 million over five years.

The inclusion of improved contraceptive coverage brings the overall incremental costs down for the provision of the same package, despite increasing costs for contraception methods. The resultant reduced pregnancies and births results in lower demand for other services in the form of the LiST interventions, and hence reduced costs in other areas.

### Costs to lives saved ratios

Achieving value for money requires that outcomes be related to the costs to achieve them. Cost per life year gained ratios can assist to indicate the relative impacts of costs and impacts on lives saved The costs per life year saved for each intervention are given in Additional file 2: Worksheets 4 and 5. The ratios demonstrate the relationship between life years saved and the costs required to achieve these life years saved, which can also be used to prioritize interventions.

Scaling up neonatal resuscitation by 55 % for example, could save 305 neonatal lives, and has a cost per life saved ratio of US$2 per life year saved. The budget impact over five years was estimated at between US$29,000 and US$33,000, depending on the degree of increase in contraceptive usage . Scaling up labour and delivery management by 5 % could save about 1,000 additional lives and has a cost per life year saved of US$420 per life year saved where contraceptive prevalence increases by 0.5 percentage points per year, and US$296 where contraceptive prevalence increases by 1 percentage point per year.

Having impact and costing information as well as a ratio between the two could enable to province to prioritize the most effective interventions, those with the least budget impact, or those with the most favourable ratio between the two.

## Discussion

This analysis represents the first attempt to undertake a prioritization and costing analysis for maternal and child health interventions at a provincial level in South Africa, taking into account local diseases burden, demographics and intervention coverage in the province, to inform the strategic planning process and ensure it is relevant to the specific needs of the KZN population. LiST has previously been described as a catalyst in programme planning in other African countries where used for in-country analyses [35]. Costing analysis is a more recent addition to the LiST, and has enabled the identification of intervention priorities in South Africa and costs at a national level, and the costs and impacts of scaling up targeted interventions on particular outcomes [36, 37]. All of these analyses have shown that a small number of interventions from the large collective of potential interventions can have substantial impacts on mortality in the different settings. The South African MDG analysis highlights that a "one size fits all" set of priorities, as the national-level analysis developed, does not address local targets for the implementation of key interventions [3]. We have shown that by prioritizing seventeen interventions plus family planning, several thousand additional neonatal, child and maternal lives could be saved and stillbirths prevented over the next five years in KZN province.

Family planning is the most significantly impactful single intervention for saving the lives of mothers and babies [38]. Family planning not only prevents additional deaths, but also reduces the overall additional costs of delivery of the priority interventions. Because increased contraceptive prevalence means fewer pregnancies and births which reduces the demand for other maternal and child health interventions in future. This could not only help free up much required resources to be used in other developmental programmes, but also potentially assist to ensure that existing programmes in interventions can be delivered at a higher level of quality, given that delivery and treatment facilities will have to deal with fewer births, complications of pregnancy and sick children. Unfortunately, the model itself is unable to capture these effects directly.

Of all the LiST interventions, labour and delivery management has the most significant impact overall through its effect on stillbirth outcomes, maternal and neonatal deaths. The impact of labour and delivery management is determined by a combination of factors including the proportion of deliveries and the level of obstetric care offered in facilities (comprehensive emergency obstetric care, basic emergency obstetric care, or essential care). Thus the impact of labour and delivery management reflects both the increase in facility births and level of care provided. Promotion of breastfeeding can potentially also make a significant contribution to saving child lives in the province. Other important interventions are largely curative, such as provision of oral rehydration solution to children with diarrhea, and full supportive care for sepsis and pneumonia in neonates.

There are additional cost considerations related to scaling up coverage for any of the interventions and we recommend that the province prioritize in order of effectiveness and cost effectiveness of the various potential interventions for saving maternal and child lives and preventing stillbirths.

In line with the South African National Treasury’s framework for strategic plans, this analysis shows the potential for health impacts of delivered programmes, as well as estimating the additional costs required to achieve these improved health outcomes to ensure value for money.

The explicit derivation of the outcomes, the costs required to achieve them and the ratios between outcomes achieved relative to costs, can empower provincial authorities to make decisions as to what interventions to prioritize, on the basis of effectiveness, budget impact or costs in relation to outcomes.

### Study limitations

While this analysis has attempted to show outcomes and costs for the next five years, there are some challenges which will need to be addressed. This analysis represents modelled outcomes of what could be achievable if coverage can be increased for key interventions. However, translation from theory into practice can be difficult, and actual real world outputs and impacts on mortality will depend on the ability of the existing system to support the increased coverage of these interventions, and to overcome implementation barriers. On the basis of this analysis, the province has implemented an auditing program for the use of partograms to monitor labour, to improve labour and delivery management. In addition a pilot project to target family planning services in one of the more deprived districts in the province has begun. The impacts and outcomes of these programmes will require monitoring and evaluation to measure their impact.

The estimations of “achievable” coverage goals for 2019 remain fairly arbitrary as it is difficult to estimate what the province might actually be capable of in the next five years if the resources are correctly deployed. Relatively high levels of coverage for interventions such as prevention of mother to child transmission (PMTCT) of HIV in the province have however shown that high coverage levels for intervention can be attained with concerted effort.

In addition, the coverage levels inputted into the model cannot take into account the quality aspects of the delivery of the interventions. So, for example, where vaccination coverage has been estimated at 72 %, it is assumed that vaccinations which are received are administered correctly and have had cold chain conditions maintained. It is also assumes the training of birth attendants and other health care workers is adequate and that a critical mass of such individuals is present in each facility, whereas a survey in South Africa has shown that there are frequently insufficient personnel at some delivery sites [39].

Cost calculations show the minimum of what would be required and do not include the costs of infrastructure development, training of staff where necessary and additional promotional activities. Taking these into account would increase the total costs. The estimates provided can however still guide provincial health department budgetary considerations with regard to providing a maternal and newborn health package.

The heterogeneity in mortality rates and intervention coverage across the province is also not adequately captured by the provincial-level analysis. KwaZulu-Natal has 11 health districts which report varying coverage levels for key interventions such as childhood vaccines, PMTCT, as well as access to utilities such as piped water. In order to completely localize the estimates from the LiST tool, an analysis would have to be completed at a district level.

## Conclusion

This modelling has attempted to identify and cost priority intervention areas for impact on maternal and child mortality over the next five years in KwaZulu-Natal Province, South Africa. We show that focusing on seventeen essential interventions and improvement of contraceptive use prevalence could result in an MMR of 129 per 100,000 live births and an NMR of 9 per 1,000 live births, and under-5 deaths of 27 per 1,000 live births. The additional cost per capita per annum for this is US$1.72. Improvement of family planning coverage saves both costs and lives. If family planning coverage and contraceptive prevalence also increase simultaneously, 2600 to 5300 additional deaths can be avoided as well as achieving significant cost savings for the system as a whole.

The priority interventions identified in this analysis are specific to the province and reflect its burden of disease and potential for improvement in coverage – additional analyses would be necessary to determine priorities for other provinces in the country.

In the post-2015 agenda, investing in family planning and decisions to prioritize either most effective or most cost effective identified interventions will be key to achieving the KwaZulu-Natal province’s outcome and financial goals.

## Additional files

Additional file 1: **Sheet 1.** Interventions added to List. Additional interventions not included in the LiST Tool, with descriptions, effect sizes and affected fractions. **Sheet 2.** Adjusted Mortality. Deaths reported to Statistics South Africa, adjusted for under-reporting to estimate actual mortality rates for neonatal and under-five mortality. **Sheet 3.** Changes in contraceptive mix. Changes in the assumption of proportion of different contraceptive choices used by the population over time (2015 to 2019). (XLSX 15 kb)

Additional file 2: **Sheet 4.** Summary of impacts of scaling up LiST interventions to achievable levels from baseline by 2019 and a 0.5 percentage point per annum increase in contraceptive prevalence. Summary of lives saved, life years gained, costs over five years and cost to life years gained ratios for all interventions where contraceptive prevalence increases by 0.5 percentage points per annum. **Sheet 5.** Summary of impacts of scaling up LiST interventions to achievable levels from baseline by 2019 and a one percentage point per annum increase in contraceptive prevalence. Summary of lives saved, life years gained, costs over five years and cost to life years gained ratios for all interventions where contraceptive prevalence increases by one percentage point per annum. (XLSX 27 kb)
